# Past landscape structure drives the functional assemblages of plants and birds

**DOI:** 10.1038/s41598-021-82851-8

**Published:** 2021-02-09

**Authors:** Lucie Lecoq, Aude Ernoult, Cendrine Mony

**Affiliations:** UMR CNRS ECOBIO, University of Rennes 1, Avenue du Général Leclerc, 35042 Rennes Cedex, France

**Keywords:** Ecology, Community ecology

## Abstract

Landscape structure is a major driver of biodiversity in agricultural landscapes. However, the response of biodiversity can be delayed after landscape changes. This study aimed to determine the effect of current and past landscape structure on plant and bird assemblages. We used a trait-based approach to understand their responses to landscape simplification and habitat fragmentation. We quantified landscape structure at three different years (1963, 1985, 2000) and sampled current plant and bird assemblages in twenty 1 km^2^ landscape windows located along the Seine Valley (France). For each window, we calculated plant and bird species richness, Community Weighted Variance (CWV), and Community Weighted Mean (CWM) of five functional traits related to dispersal capacity, reproduction, and life-cycle. We detected non-random patterns of traits for both taxa. Plant and bird species richness was lower in simple landscapes. The functional variance of plant traits was higher in landscapes simple in configuration. Both plant and bird assemblages strongly responded to past landscapes, especially their traits related to reproduction and life-cycle. It suggests that landscapes of the Seine valley will face a functional extinction debt. Further research is needed to better predict the delayed response of biodiversity expected to occur after landscape structure changes.

## Introduction

**Figure 1:**
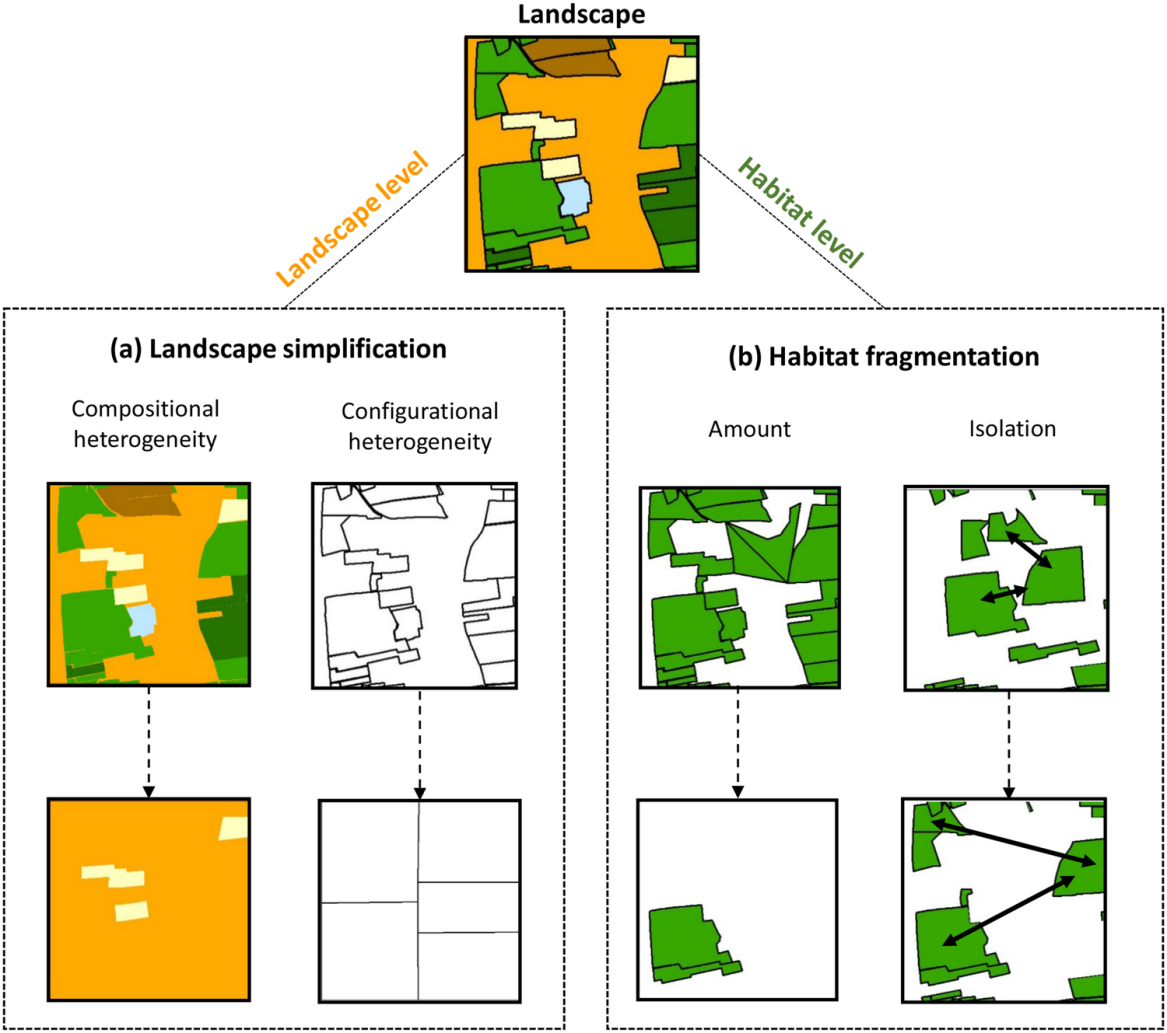
The two processes involved in the definition of landscape structure in the agricultural intensification context. (**a**) Landscape simplification is a process that is measured at the landscape level, where all classes of land use are taken under consideration. It is defined as the reduction in compositional heterogeneity (i.e. habitat diversity) and/or the reduction in configurational heterogeneity (i.e. complexity of the spatial pattern)^2^. (**b**) Habitat fragmentation is a process that is measured at the habitat level, where only one class of land use is taken into consideration. It is defined as a reduction in habitat amount and/or an increase in the isolation of habitat patches^91^. The four dotted arrows represent the direction of an increase in landscape simplification and habitat fragmentation. This figure was created using ArcGIS Software (v. 10.6.1, https://desktop.arcgis.com).

Biodiversity loss is increasing at an unprecedented rate at the global scale and agricultural intensification is known to be a major driver of this process in human-dominated regions^1,2^. Agricultural intensification is not only reflected by an increasing use of phytosanitary products and higher mechanization, but also in alteration of the structure of the landscape through two processes: landscape simplification and habitat fragmentation (Fig. 1). The alteration of the landscape structure influences the assembly of plants or animals^3–5^. At the landscape level, landscape simplification implies a decrease in both components of heterogeneity (i.e. compositional and configurational heterogeneity), generally resulting in reduced species richness within landscapes^3^. At the habitat level, habitat fragmentation implies a decrease in the amount and/or the increase of the degree of isolation of the habitat concerned^6^, generally resulting in a decrease in the richness of species specific to this habitat^7,8^. The importance of distinguishing landscape simplification and habitat fragmentation when investigating the response of biodiversity to the landscape structure has recently been underlined by the debate over their conceptualization and the appropriate scale at which they should be studied^9–11^. Therefore, it appears essential to choose gradients at both landscape and habitat level, to better understand the impact of alteration to the landscape structure on biodiversity.

Both landscape simplification and habitat fragmentation can affect biodiversity and an increasing number of studies have attempted to understand the species-related mechanisms underlying the observed response of assemblages^12–16^. In particular, using trait-based approaches offers promising avenues to better understand the effects of the landscape structure on the composition of assemblages^15,17^. The trait-based approach allows biological assemblages to be characterized no longer through distinct taxonomic groups but through continuous quantitative measurements of trait values. For example, it is possible to quantify the dispersion of trait values around the mean (e.g. Community Weighted Variance) to investigate the assembly processes driving species assemblages^14^: the decrease of the dispersion (i.e. convergence) is usually attributed to the effect of environmental filtering^18^, while an increase of the dispersion (i.e. divergence) is usually attributed to the limiting similarity^19^, or more recently to the environmental heterogeneity of microhabitats^20^. It is also possible to quantify the mean within an assemblage (e.g. Community Weighted Mean) to identify toward which optimal value the convergence occurs^21^. In a context of a filtering effect of the landscape structure, the observed assemblages might then present values of trait significantly different from randomly selected species in the regional pool: either under-dispersed trait values, indicating a convergence of strategies toward a specific trait value, or over-dispersed trait values indicating a divergence^22^. The indices based on functional traits and used to describe biodiversity aim to be independent of species richness. These indices, by taking the different characteristics of each species into account, are complementary to traditional measures of biodiversity that are based on the assumption that all species are equal facing environmental conditions^23^.

Low compositional or configurational heterogeneity has been shown to reduce functional variance (i.e. reduce the range of dispersion trait values around the mean) by selecting for specific strategies^12,16^. A decrease in heterogeneity (i.e. landscape covered by few land-use types and/or with very large patch area) in agricultural landscapes could lead to a convergence of trait values toward high dispersal potential, whether in space or time, or a high reproduction rate resulting in a greater mass effect^24^ (i.e. a higher rate of propagule influx that allows species to establish in sites where they cannot maintain viable populations). In addition, low agricultural landscape heterogeneity, reflecting intensive land management^25^, could filter traits related to the timing of reproduction. Species could be selected to reproduce before too intense disturbances occur^26^ or to synchronize their period of reproduction with resource abundance peaks to feed their offspring^27^. Habitat fragmentation can also reduce functional variance of assemblages by favouring species that are able to survive in a small patch and/or able to disperse over longer distances. In landscapes with small amounts of habitat or high isolation, species with a high dispersal capacity^28^ could be selected, especially organisms that need several patches of the same habitat throughout their lifetime (i.e. supplementation^29^). Landscape simplification and habitat fragmentation could then filter species according to specific strategies, regardless of the taxon under consideration.

Interest in the functional structure of assemblages in relation to landscape metrics is currently growing^14,30^. However, one overlooked component of the landscape is its variability over time. Indeed, landscapes are dynamic and the response of organisms to environmental changes is not necessarily immediate^31^. Assemblages can display a time-lagged response (i.e. relaxation time^32^) and can include a certain number of species predicted to become extinct (i.e. extinction debt^33^) as the assemblage reaches a new equilibrium after these environmental changes. These two processes have been identified across several taxa^34,35^ but few studies have investigated the question in the light of functional traits (but see^36–38^). The trait-based approach is a promising method to understand the mechanisms underlying relaxation time. Indeed, some functional traits could promote a delay in the organism’s response by maintaining them for a period of time before they become extinct. In plants for example, this could be a high investment in clonality as it provides the opportunity for species that are no longer able to disperse to survive at the local level over a more or less long term^39^. For birds, traits related to breeding parameters can indicate a higher sensitivity to habitat fragmentation^40^ and can affect their response delay. For instance, a longer lifespan associated with a low reproductive capacity (e.g. *Buteo buteo,* L. 1758), can imply a slower response to changes in the environment than more productive short-lived species. Analyzing the relationship between the current and past landscape structure and the distribution of traits within assemblages can help to detect delayed responses by organisms and predict the long-term effects of changes in agricultural landscapes.

This study aimed to determine the effects of current and past landscape structures on the functional assemblages of two contrasting taxa in terms of dispersal capacity: plants and birds. The study was conducted in the agricultural landscape of the Seine valley in Normandy (France), which is characterized by a gradient of landscape simplification and habitat fragmentation. Its temporal dynamics are mainly due to agricultural intensification that resulted from the implementation of the Common Agricultural Policy (CAP) in the 1960s. We quantified landscape structure at three different periods: before the implementation of the CAP (i.e. 40 years ago, 1963), after it was first implemented (i.e. 15 years ago, 1985) and after several successive reforms (i.e. current, 2000). We sampled current plant and bird assemblages in twenty 1 km^2^ square landscape windows. To extend the reach of our results, we studied plant assemblages in two different types of semi-natural habitat—grasslands and hedgerows. We first evaluated the randomness of the functional variance of assemblages through two null models: one based on the presence/absence of species (NM1), the other one on the occurrence rate of species (NM2). These two null models allowed us to highlight a possible convergence or divergence of trait values within assemblages. We then examined the independent effects of landscape simplification characterized by compositional and configurational heterogeneity (i.e. Shannon index and mean patch area respectively) and habitat fragmentation characterized by habitat amount and isolation of both plant habitat types (grassland percentage or length of hedgerows, and mean nearest distance between grassland patches or the number of disconnected networks of hedgerows; please see “Methods” for further information) on species richness, trait ranges (CWV) and mean values (CWM). To investigate the potential delayed response of plant and bird assemblages, we developed one linear model for each year (current, 15 years ago, 40 years ago). For each taxon, we focused on 5 traits related to dispersal, reproduction, and life-cycle (Table 1). Specifically, we tested the following hypotheses: (i) Functional variance of traits within plant and bird assemblages is not randomly distributed at the landscape scale; (ii) An increase of the landscape simplification and habitat fragmentation act as environmental filters and leads to lower species richness and convergence of trait values within assemblages; (iii) Functional assemblages of plants and birds are determined by past rather than present landscape structure, due to the relaxation time occurring after changes in landscape structure.

**Table 1:** Functional response traits selected for plants and birds.

Functional traits	Process	Trait value			
Description		Min	Max	Mean	SD
**Plants**					
**Seed mass (mg)**	Dispersal capacity	0.01	47.6	2.6	6
Mean seed mass					
**Allocation to clonal reproduction**		1	5	2.3	1
Index from 1 to 5, the value 5 representing a strictly vegetative reproduction					
**Beginning of flowering (month)**	Phenology	1	9	5.7	1.2
Mean value at the beginning of flowering by species					
**Flowering duration (month)**		1	12	3.6	1.7
Mean flowering duration measured by species					
**Lifespan**	Life cycle	1	4	3.3	1.2
Index ranging from 1 to 4, with the value 1 representing species with a maximum cycle of one year and the value 4 representing species with more than one generative phase in their lives					
**Birds**					
**Body mass (g)**	Dispersal capacity	8.5	10,950	331.8	1256
Mean body mass measured by species					
**Egg number**		2	12	5.6	2.1
Number of eggs per average brood per species					
**Beginning of breeding (months)**	Phenology	4	7	5.3	1
Mean value of the beginning of breeding by species					
**Brood number**		1	3.5	1.7	1
Mean number of broods per year measured by species					
**Lifespan (years)**	Life cycle	5	39	14.8	6.8
Maximum lifespan per species recorded per banding					

## Results

A total of 241 species of hedgerow plants, 173 species of grassland plants, 84 species of birds from all habitats were identified across the 20 landscape windows surveyed. In each landscape window, hedgerow plant species richness varied from 58 to 108, grassland plant species richness varied from 32 to 78, and bird species richness varied from 5 to 37.

### Non-random patterns of trait values

**Figure 2:**
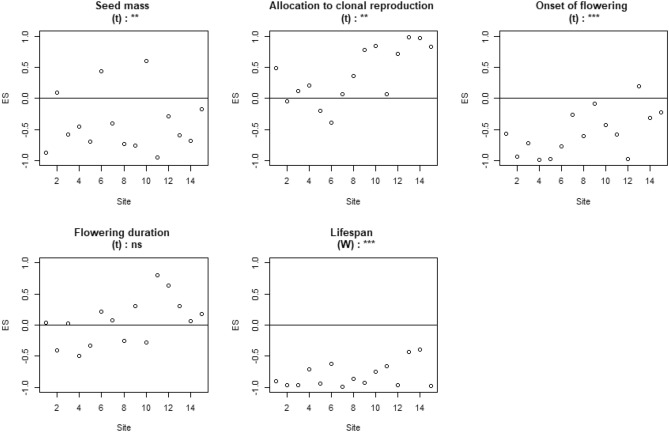
Effect size (ES) of hedgerow plant assemblages obtained using the “species occurrence rate model” (NM2) for each of the five traits: seed mass, allocation to clonal reproduction, onset of flowering, flowering duration and lifespan. Significance levels (***p < 0.001, **p < 0.01, *p < 0.05) and non-significance (ns) of the Wilcoxon tests (W) or Student’s t tests (t) are presented on top of each graph. Df = 17. This figure was created using R Software^81^ (v. 4.02, https://www.r-project.org).

**Figure 3:**
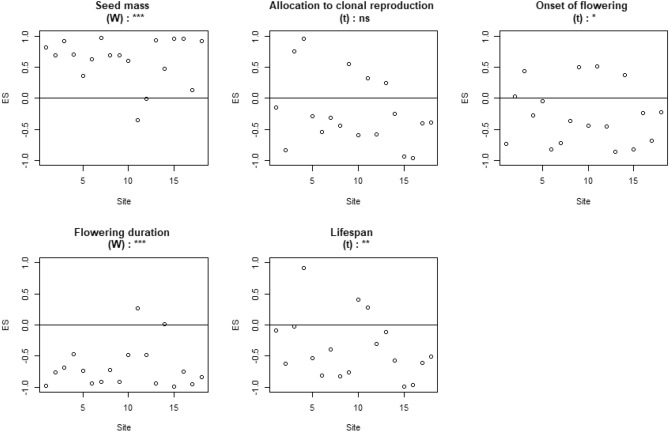
Effect size (ES) of grassland plant assemblages obtained using the “species occurrence rate model” (NM2) for each of the five traits: seed mass, allocation to clonal reproduction, onset of flowering, flowering duration and lifespan. Significance levels (***p < 0.001, **p < 0.01, *p < 0.05) and non-significance (ns) of the Wilcoxon tests (W) or Student’s t tests (t) are presented on top of each graph. Df = 14. This figure was created using R Software^81^ (v. 4.02, https://www.r-project.org).

**Figure 4:**
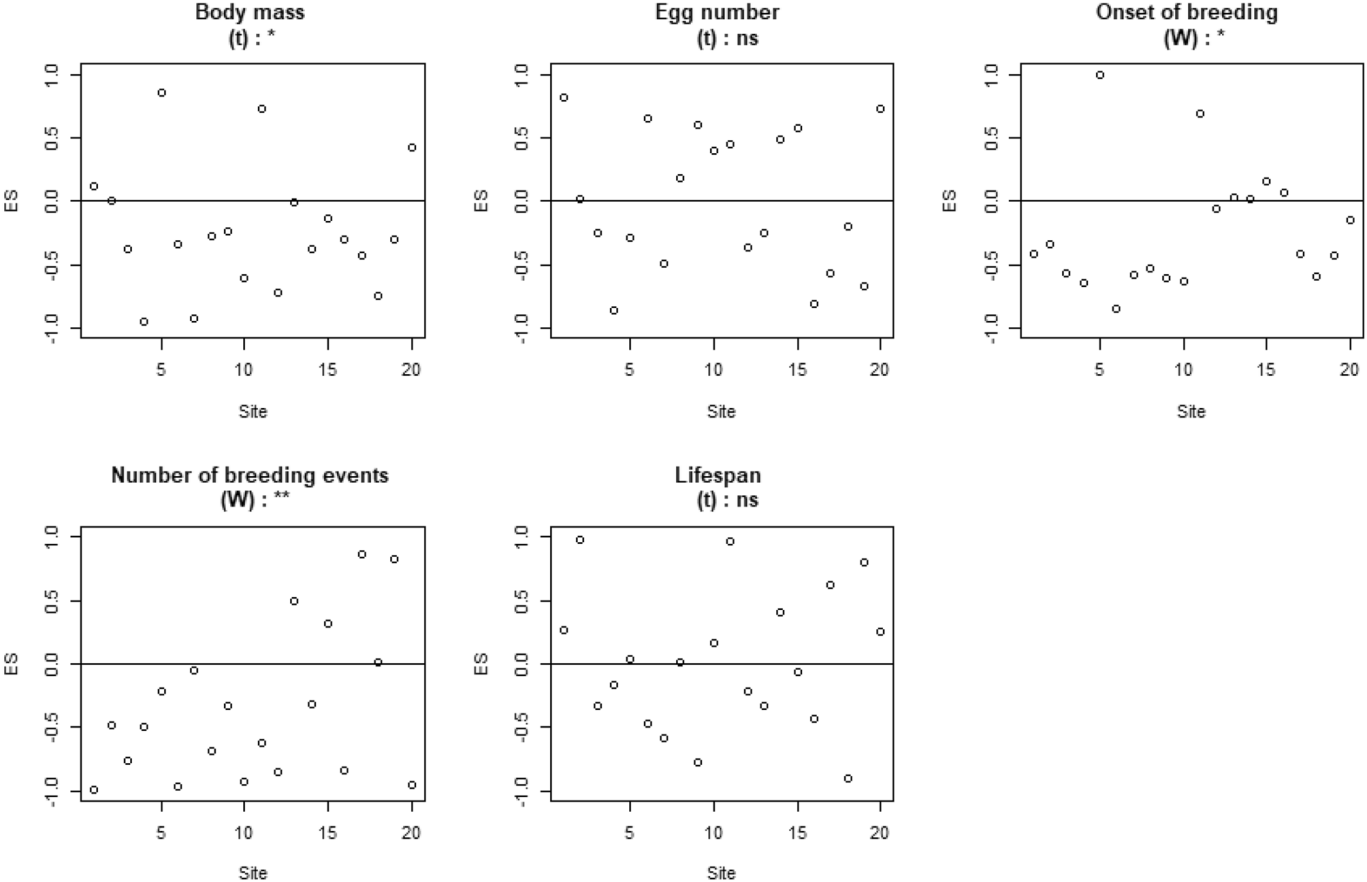
Effect size (ES) of bird assemblages obtained using the “species occurrence rate model” (NM2) for each of the five traits: body mass, egg number, onset of breeding, number of breeding events and lifespan. Significance levels (***p < 0.001, **p < 0.01, *p < 0.05) and non-significance (ns) of the Wilcoxon tests (W) or Student’s t tests (t) are presented on top of each graph. Df = 19. This figure was created using R Software^81^ (v. 4.02, https://www.r-project.org).

Our first hypothesis was that the functional variance of traits within plant and bird assemblages is not randomly distributed at the landscape scale. The two null models constructed tested if trait values influenced the presence of species (i.e. NM1), or the dominance of species within assemblages (i.e. NM2). To highlight a convergence or divergence of traits, we calculated the effect size (i.e. ES; please see “Methods” for further information): negative ES values indicate functional convergence while positive values indicate divergence. Considering the first null model based on the presence/absence of species (NM1), only one trait—flowering duration of hedgerow plant assemblages—across the three species groups (i.e. hedgerow plants, grassland plants, birds) was not randomly distributed and showed a convergence of its values (ES < 0; Supplementary Fig. S1). Considering the second null model based on the occurrence rate of species (NM2), all traits of hedgerow plant assemblages were not randomly distributed except the allocation to clonal reproduction. With the null model NM2, onset of flowering, flowering duration and lifespan showed convergence (negative ES) whereas seed mass was divergent (positive ES; Fig. 2). For grassland plant assemblages, all traits were not randomly distributed except the flowering duration. With the null model NM2, seed mass, onset of flowering and lifespan showed convergence while allocation to clonal reproduction showed divergence (Fig. 3). Within bird assemblages, body mass, onset of breeding and the number of breeding events were not randomly distributed and showed significant convergence (Fig. 4). As the null model based on the occurrence rate of species (NM2) for plants and birds showed significant results, only the community weighted variances (CWV) and community weighted means (CWM) based on species occurrence rate were tested against past and current landscape structure.

### Influence of landscape simplification and habitat fragmentation on species richness and functional assemblages of plants and birds

Our second hypothesis is that an increase of landscape simplification and habitat fragmentation act as environmental filters and leads to lower species richness and convergence of trait values within assemblages. When a trait was found to be randomly distributed following the null model NM2, the effects of the landscape structure were not tested on CWVs and CWMs. Within hedgerow plant assemblages, the species richness depended only on compositional heterogeneity: species richness was lower in landscapes with a low compositional heterogeneity (Table 2). Species richness was thus lower in simple landscapes. Considering the functional composition of these hedgerow plant assemblages, CWV of flowering duration and CWV of lifespan were higher in landscapes with a low configurational heterogeneity (Table 2). CWV of hedgerow plant assemblages for these traits were thus higher in simple landscapes. CWM of flowering duration was higher whereas CWM of lifespan was lower in landscapes with a low configurational heterogeneity (Table 2). This result indicates that simplified landscapes favour long-flowering and short-lived assemblages. Within grassland plant assemblages, species richness depended on the compositional heterogeneity and the habitat amount. Species richness was lower in landscapes with a low compositional heterogeneity or a low grassland amount (Table 3). CWV of allocation to clonal reproduction was higher in landscapes with a low configurational heterogeneity or a low grassland amount (Table 3). CWV of onset of flowering was higher in landscapes with a low grassland amount. Within grassland plant assemblages, no response of CWM was detected (Table 3). The species richness of birds was higher in landscapes with high compositional heterogeneity (Table 4). Within bird functional composition, only one trait out of the four non-random traits depended on landscape variables. CWV of number of breeding events was lower in landscapes with a high hedgerow amount. The CWM of this trait was higher in landscapes with higher grassland isolation.

**Table 2:**
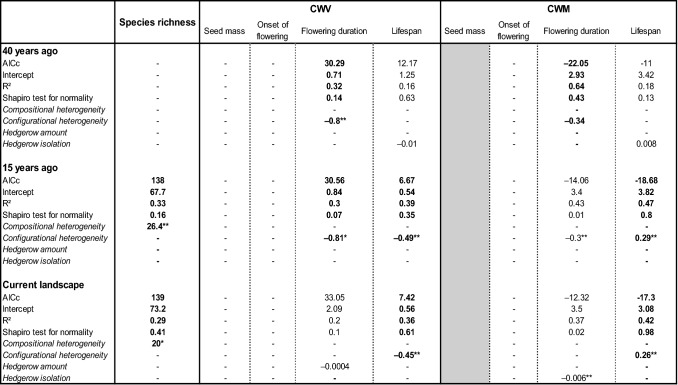
Results of linear models testing the effect of landscape predictors on species richness, community weighted variance (CWV) and community weighted mean (CWM) of each trait, calculated from the occurrence rate of plant assemblages in hedgerows.

AICc, intercept, R^2^ and Shapito test for normality of residuals of each significant model are indicated. Estimates of the landscape predictors and their significance levels (***p < 0.001, **p < 0.01, *p < 0.05) are indicated. Non-significance of models and landscape predictors (–) are indicated. Selected best-fitting models meeting the conditions of lowest AICc (ΔAICc < 2) and normal residuals are indicated by bold font. Models in normal font are included to illustrate the ΔAICc between significant models. The trait ‘allocation to clonal reproduction’ was not considered because the CWVs were found randomly distributed with the null model NM2. The trait ‘seed mass’ in the CWM section is colored in grey because CWMs for this trait were not calculated as the CWV was found divergent with the null model NM2.

**Table 3:**
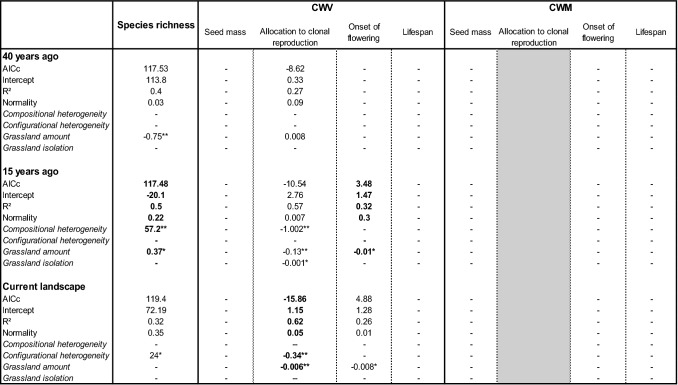
Results of linear models testing the effect of landscape predictors on species richness, community weighted variance (CWV) and community weighted mean (CWM) of each trait, calculated from the occurrence rate of plant assemblages in grasslands.

AICc, intercept, R^2^ and Shapito test for normality of residuals of each significant model are indicated. Estimates of the landscape predictors and their significance levels (***p < 0.001, **p < 0.01, *p < 0.05) are indicated. Non-significance of models and landscape predictors (–) are indicated. Selected best-fitting models meeting the conditions of lowest AICc (ΔAICc < 2) and normal residuals are indicated by bold font. Models in normal font are included to illustrate the ΔAICc between significant models. The trait ‘flowering duration’ was not considered here because the CWVs were found randomly distributed with the null model NM2. The trait ‘allocation to clonal reproduction’ in the CWM section is colored in grey because CWMs for this trait were not calculated as the CWV was found divergent with the null model NM2.

**Table 4:** Results of linear models testing the effect of landscape predictors on species richness, community weighted variance (CWV) and community weighted mean (CWM) of each trait, calculated from the occurrence rate of bird assemblages.

	Species richness	CWV			CWM		
		Body mass	Onset of breeding	Number of breeding events	Body mass	Onset of breeding	Number of breeding events
**40 years ago**							
AICc	**130.3**	–	–	**− 57.5**	–	–	**− 46.7**
Intercept	**15.2**	–	–	**0.3**	–	–	**1.94**
R^2^	**0.47**	–	–	**0.5**	–	–	**0.3**
Shapiro test for normality	**0.23**	–	–	**0.5**	–	–	**0.71**
Compositional heterogeneity	17.4***	–	–	**− 7.43**	–	–	–
Configurational heterogeneity	–	–	–	–	–	–	–
Hedgerow amount	–	–	–	− 1.1e−05**	–	–	–
Hedgerow isolation	–	–	–	–	–	–	–
Grassland amount	–	–	–	–	–	–	–
Grassland isolation	–	–	–	–	–	–	0.001**
**15 years ago**							
AICc	–	–	–	− 58	–	–	–
Intercept	–	–	–	0.3	–	–	–
R^2^	–	–	–	0.47	–	–	–
Shapiro test for normality	–	–	–	0.01	–	–	–
Compositional heterogeneity	–	–	–	–	–	–	–
Configurational heterogeneity	–	–	–	–	–	–	–
Hedgerow amount	–	–	–	**− **1.6e−05***	–	–	–
Hedgerow isolation	–	–	–	–	–	–	–
Grassland amount	–	–	–	–	–	–	–
Grassland isolation	–	–	–	–	–	–	–
**Current landscape**							
AICc	–	–	–	**− **55	–	–	**− **43.8
Intercept	–	–	–	0.3	–	–	2.04
R^2^	–	–	–	0.39	–	–	0.2
Shapiro test for normality	–	–	–	0.12	–	–	0.17
Compositional heterogeneity	–	–	–	–	–	–	–
Configurational heterogeneity	–	–	–	–	–	–	–
Hedgerow amount	–	–	–	**− **1.3e−05**	–	–	–
Hedgerow isolation	–	–	–	–	–	–	–
Grassland amount	–	–	–	–	–	–	**− **0.001*
Grassland isolation	–	–	–	–	–	–	–

AICc, intercept, R^2^ and Shapito test for normality of residuals of each significant model are indicated. Estimates of the landscape predictors and their significance levels (***p < 0.001, **p < 0.01, *p < 0.05) are indicated. Non-significance of models and landscape predictors (−) are indicated. Selected best-fitting models meeting the conditions of lowest AICc (ΔAICc < 2) and normal residuals are indicated by bold font. Models in normal font are included to illustrate the ΔAICc between significant models. The traits ‘egg number’ and ‘lifespan’ were not considered here because the CWVs were found randomly distributed with the null model NM2.

### Influence of past and current landscape structure on species richness and functional assemblages of plants and birds

Our third hypothesis is that species richness and functional assemblages of plants and birds are determined by the past rather than present landscape structure, due to the relaxation time occurring after changes in landscape structure. Species richness of hedgerow plant assemblages responded to the current landscape structure and the landscape structure observed 15 years ago (Table 2). Within these hedgerow plant assemblages, CWV of flowering duration responded to the landscape structure observed 15 years ago and 40 years ago. CWM of flowering duration responded only to the landscape structure observed 40 years ago. CWV and CWM of lifespan responded to the current landscape structure and the landscape structure observed 15 years ago (Table 2). Species richness of grassland plant assemblages only responded to the landscape structure observed 15 years ago (Table 3). Within these grassland plant assemblages, CWV of allocation to clonal reproduction responded to the current landscape structure whereas CWV of onset of flowering responded to the landscape structure observed 15 years ago. Therefore, plant assemblages found in hedgerows and grasslands mostly responded to the current landscape structure (4 significant relations) and the landscape structure observed 15 years ago landscapes (6 significant relations) rather than the landscape structure observed 40 years ago (2 significant relations). Species richness, CWV and CWM of the number of breeding events of bird assemblages only responded to the landscape structure observed 40 years ago (Table 4).

## Discussion

### Functional strategies can determine the dominance of species at the landscape scale

To test our first hypothesis, we investigated the distribution of the functional variance of traits within plant and bird assemblages. We demonstrated that most traits were not randomly distributed for both plant and bird assemblages at the landscape scale when the occurrence rate of species was shuffled (i.e. NM2) rather than when species identity was modified (i.e. NM1). This suggests that trait values do not influence the presence of species but their dominance within assemblages. This result is interesting to highlight because it is recognised that the trait values of dominant species influence the ecosystem functioning (i.e. mass-ratio hypothesis^41^). Therefore, functional strategies of species at the landscape scale will not only determine their dominance within assemblages but will also influence the ecosystem functioning^14^. We demonstrated that most of these functional traits displayed convergence in both taxonomic groups. At the landscape scale, one of the first processes expected to drive the species assembly is dispersal^8,42^. The convergence of dispersal-related traits within our assemblages thus demonstrated this paradigm and validated that these traits must be taken into account in landscape-scale studies. However, two functional traits within plant assemblages showed a divergence: seed mass of plant species in hedgerows and allocation to clonal reproduction of plant species in grasslands. These two traits are also considered as traits related to the competition process^43,44^. Competition, theoretically acting on species assembly at a more local scale than dispersal^42^, can lead to more local convergences within assemblages. The multitude of local convergences gives rise to patterns of divergence, which is in agreement with previous studies that demonstrated similar mixed patterns of convergence and divergence at the landscape scale (e.g.^14,20^). We believe that further work is needed to clearly identify the key traits that mediate species response at the landscape scale by including a wider range of traits related to all stages of the regeneration-cycle of organisms^45^, such as plant establishment or bird resource-acquisition^14,16^.

### Landscape simplification and habitat fragmentation

According to our second hypothesis, higher landscape simplification and higher habitat fragmentation leads to lower species richness and convergence of trait values within assemblages. This hypothesis was verified for the species richness of both plants and birds: species richness of hedgerow plants, grassland plants, and birds were lower in landscapes with low compositional heterogeneity, which is in agreement with the results of previous studies^46–49^. Indeed, low compositional heterogeneity, which corresponds to low diversity and/or evenness in land-uses, hinders the coexistence of both species linked to a specific habitat, and generalist species which are generally the ones benefiting from landscape simplification^50^. In addition, the species richness of grassland plants was higher in landscapes with a high grassland percentage. It supports the recent habitat amount hypothesis^51^ suggesting that an increase in habitat amount in a given landscape window increases species richness. This increase is attributed to the sample area effect (i.e. larger sample areas generally contain more species^52^) and to the increase in the colonization rate between patches^19^. However, contrary to our expectation, a higher landscape simplification did not lead to the convergence of trait values within assemblages: in landscapes with simple spatial configuration, the functional variance of plant assemblages was higher, especially for plant assemblages observed in hedgerows. A low configurational heterogeneity decreases the probability of hedgerows being located at interfaces among different land-use types^2^. The reduced interfaces between hedgerows and other land-uses limits the edge effect by reducing the probability of colonization by species and can impact *in fine* the competitive hierarchies among plant species^45^. Therefore, in a simplified landscape, the reduced competition with incoming species could allow hedgerow plant assemblages to relax the convergence toward a specific functional strategy and thus to present higher functional variances.

Furthermore, we demonstrated that higher habitat fragmentation did not lead to the convergence of trait values either: the functional variance of grassland assemblages was higher in landscapes with low grassland amount. This result is not due to an indirect effect of higher species richness as we previously demonstrated that species richness was lower in this type of landscape. It means that in landscapes with a lower habitat amount, fewer plant species were present in grasslands but they presented more variable trait values. This result can be explained by the characteristics of the study area: in this agricultural but preserved study area, landscapes with a lower grassland amount were not characterized by homogeneous intensive agricultural practices as usually described in fragmented European agrosystem^53^ but by more diverse, heterogeneous practices (e.g. pasture, mowing). This diversity of local agricultural practices can lead to the selection of local trait syndromes^54^ which resulted in higher functional variance of trait values related to the phenology (i.e. onset of flowering) or local competition (i.e. allocation to clonal reproduction) at the landscape scale.

Finally, within bird assemblages only the number of breeding events was influenced by the landscape structure: the functional variance of this trait was lower in landscapes with higher hedgerow amount. In landscapes with higher hedgerow amount, bird communities may be more represented by birds specialized in this type of wooded habitat, like greenfinches *Carduelis chloris* or dunnocks *Prunella moduralis*^55^, that have a high number of breeding events (2 and 2.5 respectively whereas the mean of the regional pool is 1.7). Wooded environments may have specific constraints related to the type of resources^56^ which might have repercussions on the number of breeding events. Landscapes with higher hedgerow amount thus favour species with similar values of this trait which resulted in lower functional variance. However, this convergence did not seem to occur toward one specific strategy as no significant effect of the hedgerow amount on CWMs of this trait was detected. We demonstrated that including variables at both landscape level (compositional and configurational heterogeneity) and habitat level (habitat amount and isolation) can allow to highlight different mechanisms. However, the correlation between our variables was sometimes high which could have hidden some relations between the landscape structure and the functional assemblages. Future research should investigate this type of question using a large-scale sampling design that minimizes a priori the correlation between variables. This approach could help to disentangle the independent effect of landscape simplification and habitat fragmentation still widely discussed today^9–11^.

### The plant and bird functional traits are related to the past landscape structure

As predicted in our third hypothesis, taxonomic and functional structures of both plant and bird assemblages were driven by past landscape structure. Plants responded to both current and past landscapes whereas birds only responded to past landscapes. The time-lagged response, known as relaxation time^32^, has been demonstrated for species richness and composition of assemblages (see review of Kuussaari et al.^31^) and is also highlighted here with the delay found for species richness. However, only a few studies investigated this question using a trait-based approach (but see^16,37^). Within the studied functional assemblages, responses to past landscapes were not related to organism dispersal capacity but rather to the reproduction and the organism's life-cycle. Our study thus provides new empirical evidence for the paradigm suggesting that delayed extinctions are especially linked to the characteristics of organisms related to turnover rates^31^. Indeed, both theoretical and empirical studies suggested that delayed extinctions are more likely to occur in assemblages represented by species with low turnover rates (e.g. ^31,36,57^), mostly because they display slower responses to environmental changes^36,58^. Furthemore, as highlighted by Figueiredo et al.^59^, the plants’ level of allocation to clonality can also delay extinctions, which was demonstrated in our assemblages of grassland plants. Bird response was even more delayed than plant response within our assemblages: bird assemblages responded to the landscape structure observed 40 years ago whereas plant assemblages mostly responded to the current landscape structure and the landscape structure observed 15 years ago. This delayed response of birds may be due to two reasons. First, bird assemblages in our study area have a longer mean lifespan than herbaceous plants: 14 years and less than 5 years respectively. According to Kuussaari et al.^31^, long-lived species are likely to show slower relaxation to a new equilibrium than short-lived species. Our results are therefore consistent with this theory and with the results of other studies conducted across several taxa highlighting a long relaxation time in long-lived species^60^. Secondly, bird delayed response could also result from high plasticity in their behavior^61^. Indeed, some species can increase their territory and their food search radius to compensate for habitat loss^62^, or can adapt to unfamiliar food sources when there are alternative habitats in the surroundings if increasing mobility is too costly^63^. This behavioral plasticity thus allows them to survive for some time in an unfavorable environment before going extinct.

Beyond the interest in understanding the assembly rules of biodiversity at the landscape scale, our findings may also be useful in the context of landscape management. Indeed, we highlighted that bird assemblages still responded to the landscape structure observed 40 years ago, i.e. before the implementation of the Common Agricultural Policy (CAP). Though, this policy implemented at the European scale has profoundly changed the structure of agricultural landscapes in the last 50 years^64^. Our results therefore suggest that the extinction debt of bird assemblages has not yet been paid following the implementation of the CAP and that more impacts are expected to come in the future. Moreover, the trait-based approach validates that the delayed response of biodiversity can depend on specific species’ characteristics and that, beyond the number of species that are expected to be lost, agricultural landscapes of the Seine valley could also face a "functional extinction debt"^65^. In the current context of biodiversity loss and the associated ecological functions, it is thus important to emphasize that the effect of landscape changes, whether expected to be harmful (i.e. extinction debt^33^) or beneficial (i.e. immigration credit^66^) for biodiversity, will not be visible immediately. Moreover, the difference in response delay between the plant assemblages (primary producers) and bird assemblages (herbivorous and predatory vertebrates) can exacerbate this problem by generating temporal mismatches between trophic levels^16^. These trophic mismatches already observed in response to climate change^67,68^ can occur in response to changes in landscape structure over a longer time scale and could *in fine* alter the entire ecosystem functioning. We believe that additional empirical studies as presented here are needed and that, combined with simulation‐based investigations (e.g.^69^), they could be used to predict delayed response of biodiversity expected to occur after perturbations or restoration measures.

## Conclusion

This paper provides new insights into the response of organisms to past and current landscape structure. First, we demonstrated that patterns of convergence and divergence of traits coexist at the landscape scale as suggested by previous studies^20^. Second, we highlighted an effect of landscape structure on functional traits, especially those related to reproduction and life-cycle. These functional responses are not due to changes in species richness, indicating a direct filter effect of the landscape on the functional structure of assemblages. Third, we highlighted the major importance of the temporal component of the landscape: both plants and birds responded to past landscapes, suggesting that the biodiversity of the Seine Valley alluvial plain is still in a transitional state following the CAP implementation. Relaxation time should therefore be considered as a key process in the context of landscape management and should be taken into account when considering the long-term consequences of land-use changes. The trait-based approach can be particularly useful to identify traits that make species sensitive to response delay and thus can help to better predict the consequences of land management policies for biodiversity in the current context of global change.

## Methods

### Study site and sampling design

The study was carried out on the alluvial plain of the Seine valley in Normandy (France) and relies on the biological data collected by Ernoult et al.^70^ in 2003. This region is characteristic of the enclosed *bocage* landscape found in north-western Europe^71^. Agricultural activities are restricted mainly by floods linked to the sub-surface alluvial groundwater along the Seine^70^. Twenty (1 km × 1 km) landscape windows were specifically selected to represent the range of landscape simplification and habitat fragmentation found within the Seine valley. In the 20 landscape windows, we characterized landscape parameters both at the landscape and habitat level. To take landscape dynamic into account, we analyzed these parameters at three distinct years selected based on the introduction of the common agricultural policy (CAP), which mainly consisted of an increase in field areas by merging sets of small fields into larger fields to adapt to the use of mechanical machinery: before (1963), after it was first implemented (1985) and after several successive reforms (2000). We thus analyzed three sets of land-cover data representing: (i) the current landscape structure, i.e. observed in 2000, (ii) the landscape structure observed 15 years ago, i.e. in 1985, (iii) the landscape structure observed 40 years ago, i.e. in 1963. Major changes in land-uses within the study area took place between 1963 and 1985, with a major conversion of grasslands to crops and a loss in the total hedgerow length (Supplementary Fig. S2).

### Landscape structure analysis

Land-use within each of the 20 landscape windows was characterized using aerial photographs. We identified seven main land-use types: grasslands, hedgerows, crops, fruit orchards, artificial surfaces (i.e. roads, buildings), woodlands and water bodies. Land-use types and hedgerow networks were identified from black and white IGN (i.e. French national institute of geographical and forestry information) aerial photographs (1/20,000) for 1963 and 1985, and from colour IGN aerial photographs (1/25,000) for 2000 (Supplementary Fig. S3). The landscape structure in each of the 20 landscape windows was characterized at the three years studied. Landscape simplification was characterized by the landscape compositional and configurational heterogeneity. Shannon diversity index was chosen as a measure of the compositional heterogeneity^72^. This index integrates both the diversity of land-use types present within each landscape window and their evenness. A high value indicates a high number of land-uses and/or an even distribution of land-uses within the landscape. A value of 0 indicates that the landscape is constituted of a single land-use type. This index is independent from the patches spatial configuration (Supplementary Tables S1 and S2). In the other sections of this manuscript, we referred to this index as the compositional heterogeneity. The mean patch area was chosen as a measure of the configurational heterogeneity. This index characterizes the complexity of the spatial pattern of all land-use patches within each landscape window^2^. A small value represents a high configurational heterogeneity. To reduce skewness, this indice was log-transformed prior to any analysis. In the other sections of this manuscript, we referred to this index as the configurational heterogeneity. Therefore, the sign of this index has been reversed, as a small value of mean patch area represents a high configurational heterogeneity. Habitat fragmentation was characterized based on two specific plant habitat types: hedgerows and grasslands^73^. We selected the amount of habitat and the degree of isolation between patches. Habitat amount was measured as the percentage of the landscape window occupied by grasslands, and as the total length of hedgerows. In the other sections of this manuscript, we referred to these indices as the grassland amount and the hedgerow amount respectively. Habitat isolation of grasslands was quantified by the mean nearest Euclidean distance between grassland patches. The isolation of hedgerows was quantified by the number of disconnected networks of hedgerows. In the other sections of this manuscript, we referred to these indices as the grassland isolation and the hedgerow isolation respectively. All indices were calculated using Fragstat software^72^, except total length and the number of hedgerow networks that were calculated using the ‘calculate geometry tool’ in ArcGis software. Correlations (Spearman) between landscape variables were under 0.87 at each year (Supplementary Tables S1 and S2).

### Biodiversity surveys

Within each landscape window, we surveyed plant assemblages (floristic surveys of herbaceous plants in hedgerows and grasslands) and bird assemblages (point count data). Among the plant groups, we studied two independent habitats: grasslands and hedgerows. Biological data were sampled in one session by Ernoult et al.^70^ in 2003. Within each landscape window, from 12 to 27 floristic surveys of grasslands were conducted, corresponding to 294 surveys, due to the absence of grasslands in five landscape windows and inaccessible fields in other windows. In each sampled grassland, a 4 m x 4 m plot was delimited at the center of the patch and divided into 16 quadrats. In each quadrat, a sample of 0.20 × 0.20 m was conducted (Supplementary Fig. S4). Within each landscape window, from 8 to 20 floristic surveys of hedgerows were conducted, corresponding to 274 surveys, due to the absence of hedgerows in 2 landscape windows and inaccessible hedgerows in other windows. In each sampled hedgerow, a 10 m^2^ section was selected and divided into 10 quadrats of 1 m × 1 m on each side of the hedgerow (Supplementary Fig. S4). Plant species were identified and their abundance was defined as the number of occurrences in each plot. Within each landscape window, four bird surveys spaced 500 m apart were carried out with a total of 80 sampling points. Bird surveys were conducted in May and June 2003 using the point-count method^74^: all nesting species from any land-uses detected visually and acoustically over a 20-min listening period were recorded. To study bird and plant assemblages at the landscape-scale diversity, all surveys were pooled per landscape window (Supplementary Table S3, Supplementary Figs. S4 and S5). For both birds and plants, the analyses of these data were based on the presence/absence of species, and on the occurrence rate of species.

### Functional traits and indices

Five functional response traits were selected for plants and birds to reflect key functional aspects of their life-history: dispersal, reproduction and life-cycle (Table 1). For plants, values were extracted from the Biolflor^75^ and LEDA^76^ databases. For birds, trait values were collected in Duquet^77^. In rare cases (< 2% of the species analyzed) when data were not available at the species level, trait values were determined by calculating the mean trait value of species belonging to the same genus. For species with several values for the same trait, the mean of the available data was calculated. To avoid redundancy in our analysis, we checked that trait correlations were under 0.7^78^. Traits were at most correlated with a Spearman coefficient of 0.7 (Supplementary Table S4).

For each trait, we calculated two functional indices: the community weighted mean (CWM) and the community weighted variance (CWV) to characterize independently the functional structure of each biological model. These functional indices were measured for each landscape window. First, we quantified CWM which corresponds to the mean value of the functional trait considered, weighted by the relative abundances of the different species in each site^79^. Secondly, we calculated the functional variance within communities as CWV to quantify the deviation of trait values from the mean^80^. These two indices are defined as:

$${CWM}_{jk}= \sum_{i=1}^{n}{P}_{ik}\times ({trait}_{ij})$$

$${CWV}_{jk}= \sum_{i=1}^{n}{P}_{ik}\times {\left({trait}_{ij}\right)}^{2}- {\left(CWM_{jk}\right)}^{2}$$

where n is the total number of species in site k, P_ik_ is the presence/absence or the occurrence rate of species i in site k and trait_ij_ is the value of trait j for species i. CWV and CWM were calculated using R software^81^.

### Data analysis

We first analyzed trait phylogenetic signals to ensure that the information obtained in our analyses was not related to the evolutionary history of the species. The phylogenetic signal is defined as the tendency of related species to resemble each other more than randomly selected species in the phylogenetic tree^82^. This signal was tested for each functional trait using Blomberg’s K^83^ with the phylogenetic tree of Zanne et al.^84^ for plants, and the tree of Jetz et al.^85^ for birds using the "phytools" package^86^. Of all the species sampled, 82% of plants and 98% of birds were included in phylogenetic trees. All the functional traits of plant species (except flowering duration) and bird species showed a significant phylogenetic signal (Supplementary Table S5). However, the phylogenetic signals of the traits studied were lower than expected in a Brownian model (K < 1^45^). Furthermore, as our question focused on the landscape filtering effect operating at the human time scale and not at the scale of an evolutionary process, phylogenetic information was not included in statistical models as recommended by de Bello et al.^87^.

Our first hypothesis is that the functional variance of traits within plant and bird assemblages is not randomly distributed at the landscape scale. To test this hypothesis, the observed CWV values of each assemblage were compared to the values of the same index calculated from random assemblages. This method tested for a possible convergence of trait values towards a single strategy (i.e. landscape filter effect) or a possible divergence of strategies (i.e. landscape-scale divergence or local convergences): if the observed assemblages present values of trait significantly under-dispersed, it indicates a convergence of strategies toward a specific trait value; if the observed assemblages present values of trait significantly over-dispersed trait values it indicates a divergence^22^. Thus, two null models (adapted from the scripts of Bernard-Verdier et al.^88^) were constructed: one to test if trait values influence the presence of species, and the second to test if trait values influence the dominance of species within assemblages. The species selection model (NM1) was based on the presence-absence of species in each assemblage and aimed to test the null hypothesis that species identity is randomly distributed from the regional species pool. In this first model, only the identity of the species was modified, the total species richness of each assemblage was fixed. The probability of a species to be drawn was weighted by its relative abundance in the regional pool (i.e. abundant species have a higher probability of being drawn than rare species). The species occurrence rate model (NM2) was based on the species occurrence rate and aimed to test the null hypothesis that the proportion of each species within assemblages is random. In this model, the species richness and identity of each species remained fixed, while occurrence rate values of species within each landscape window were randomly shuffled. For each biological models (i.e. hedgerow plants, grassland plants, and birds), 5 traits and 2 null models, 999 null assemblages were simulated and CWV was calculated for each. To highlight a convergence or divergence of traits, the observed CWV values were compared to those under the null hypothesis via the calculation of the effect size (i.e. ES). ES was preferred to Standardized Effect Size (SES) due to the non-normal distribution of null values^88^. The ES is defined as:

$$ES=\left(\frac{ Number \left(null<obs\right)+ \frac{Number\left(null=obs\right)}{2}}{1000}-0.5\right)\times 2$$

ES varies between − 1 and 1: negative ES values indicate functional convergence while positive values indicate divergence. For each trait and model, we used Student’s t-tests (when distributions were normal) and Mann–Whitney tests (when distributions were non-normal) to test the significant difference of ES compared to zero.

When the CWV of the assemblages differed from random, we tested our second hypothesis: we expected that landscape simplification (i.e. decrease in compositional and configurational heterogeneity) and habitat fragmentation (i.e. decrease in habitat amount and increase in isolation) act as environmental filters and lead to the convergence of trait values within assemblages. We thus analyzed the effect of landscape variables on the CWV. The observed CWVs were preferred over ES because the models had better coefficients of determination, the results of the two methods being relatively similar. Furthermore, when null models showed a convergence of values for a given trait, the CWMs were also studied in response to landscape variables following the same models as CWV. In addition, to have keys of understanding concerning possible indirect functional responses related to taxonomic richness (i.e. increase in functional variance due to an increase in species richness), we analyzed the effect of landscape variables on the species richness of each taxon. Very few CWVs were significantly correlated with species richness (Supplementary Table S6). For plant assemblages, we performed linear regression with four explanatory variables. For both hedgerow plant and grassland plant assemblages, the variables describing landscape simplification were the compositional heterogeneity and the configurational heterogeneity. At the habitat level, we included (i) the hedgerow amount and isolation for hedgerow plant assemblages and (ii) the grassland amount and isolation for grassland plant assemblages, as variables describing habitat fragmentation. For bird species assemblages, we performed linear regression with the six above explanatory variables because the sampling of assemblages was not focused on a single habitat type. The percentage of crops was not included in these regressions as it was correlated with the grassland amount over 0.60 (Spearman test; Supplementary Table S7).

To test our third hypothesis that functional assemblages of plants and birds depend on past landscape structure rather than present landscape structure, we relied on the methodology conventionally used in articles investigating the response of organisms to the past: we confronted current biological data with past and current landscape variables. However, due to the important multicollinearity with all predictors included in a single model per trait (Supplementary Table S8), we choose to develop one model for each year (current, 15 years ago, 40 years ago). For each trait, the best model was selected by the Akaike information criterion with correction for small sample size (AICc^89^). We selected the best-fitting model with lower AICc (ΔAICc < 2). All analyses were performed using R software with the 'MASS'^52^, and 'car'^90^ packages.

## Supplementary Information

Supplementary Information.

## Data Availability

The datasets analysed during the current study are available from the corresponding author on reasonable request.
