# Supplementary material for: Past landscape structure drives the functional assemblages of plants and birds
Source: Sci Rep. 2021 Feb 9;11:3443. doi: 10.1038/s41598-021-82851-8 (PMC7873185; doi:10.1038/s41598-021-82851-8)
Supplement: Supplementary file 1 — Supplementary Information. [file 41598_2021_82851_MOESM1_ESM.pdf]

Supplementary Information

Past landscape structure drives the functional assemblages of plants and birds

Lecoq L.[1], Ernoult A. [1], Mony C.[1]

[1]: UMR CNRS ECOBIO, University of Rennes, Avenue du Général Leclerc, 35042 Rennes Cedex, France

Table

Supplementary Figure S1: Results of the null models NM1 of each biological model

Supplementary Figure S2: Percentages of land-uses and length of the hedgerow network of each year within the study area 20 landscape windows along the Seine valley

Supplementary Figure S3: Aerial images of one landscape window at each year (1963, 1985, 2000)

Supplementary Table S1: Results of Spearman tests (coefficient and p-value) between landscape variables of each year used in linear models for hedgerows and grasslands

Supplementary Table S2: Results of Spearman tests (coefficient and p-value) between landscape variables of each year used in linear models for birds

Supplementary Figure S4: Protocol of biological surveys conducted in the Seine valley in 2003.

Supplementary Table S3: List of all plant and birds species found across the 20 landscape windows along the Seine valley

Supplementary Figure S5: Rarefaction curves of hedgerow and grassland assemblages

Supplementary Table S4: Results of Spearman tests (coefficient and p-value) on functional traits of hedgerows, grasslands and birds

Supplementary Table S5: Significance of the phylogenetic signals of the 5 continuous traits of plants and birds

Supplementary Table S6: Results of Spearman tests (coefficient and p-value) between species richness and CMVs of each biological model

Supplementary Table S7: Results of Spearman tests (coefficient and p-value) between percentage of grasslands and percentage of crops for the three years

Supplementary Table S8: Results of the variance inflation factors (VIFs) of all linear models

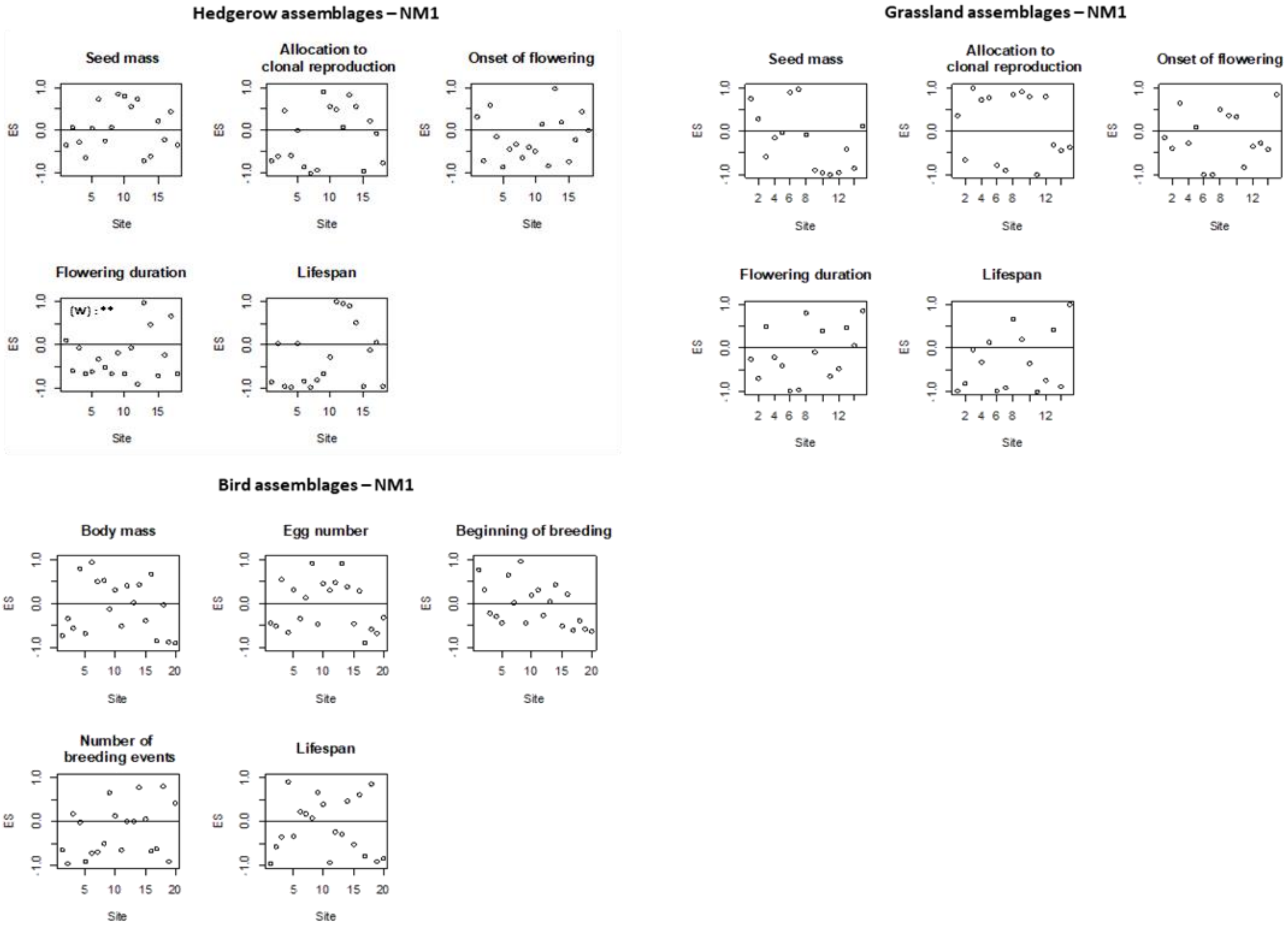

Supplementary Figure S1: Results of the null models NM1 of each biological model

The species selection model (NM1) was based on the presence-absence of species in each assemblage and aimed to test the null hypothesis that species identity is randomly distributed from the regional species pool. In this first model, only the identity of the species was modified, the total species richness of each assemblage was fixed. The probability of a species to be drawn was weighted by its relative abundance in the regional pool (i.e. abundant species have a higher probability of being drawn than rare species). Significance level (**p<0.01) of the only significant Wilcoxon test (W) is presented on the figure of flowering duration of hedgerow assemblages. This figure was created using R Software (v. 4.02, URL: https://www.r-project.org).

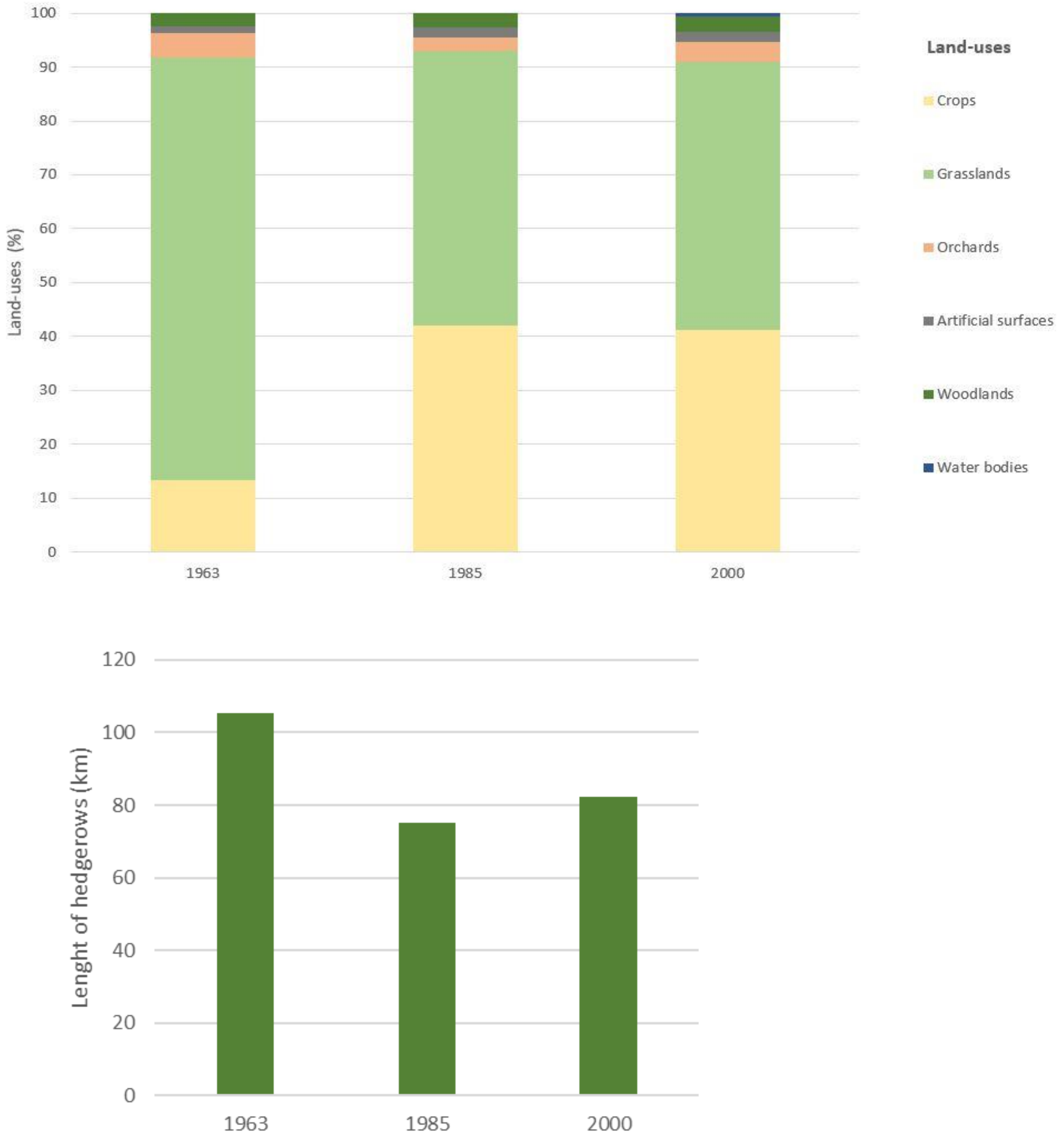

Supplementary Figure S2: Percentages of land-uses (up) and total length of the hedgerow network (down) of each year within the study area (20 sites along the Seine valley).

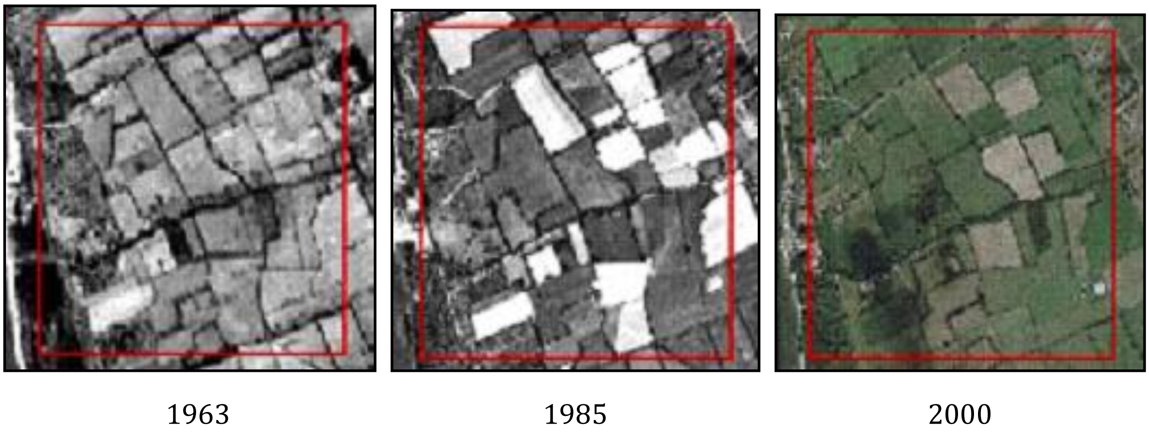

Supplementary Figure S3: Aerial images of one landscape window at each year (1963, 1985, 2000). This figure was created using ArcGIS Software (v. 10.6.1, URL: https://desktop.arcgis.com). Aerial images were bought in 2002 from the French National Institute of Geographic and Forestry Information (IGN, URL: https://www.ign.fr/).

Supplementary Table S1: Results of Spearman tests (coefficient and p-value) between landscape variables of each year used in linear models for hedgerows and grasslands

**Hedgerows**								
2000	Mean patch area		Shannon diversity index		Hedgerow length		Number of disconnected networks of hedgerows	
	Coefficient	p	Coefficient	p	Coefficient	p	Coefficient	p
Mean patch area	1	0						
Shannon diversity index	-0.85	<0.001	1	0				
Hedgerow length	-0.76	<0.001	0.61	0.007	1	0		
Number of disconnected networks of hedgerows	-0.81	<0.001	0.67	0.002	0.8	<0.001	1	0
1985	Mean patch area		Shannon diversity index		Hedgerow length		Number of disconnected networks of hedgerows	
Mean patch area	1	0						
Shannon diversity index	-0.87	<0.001	1	0				
Hedgerow length	-0.74	<0.001	0.67	0.002	1	0		
Number of disconnected networks of hedgerows	-0.75	<0.001	0.62	0.005	0.75	<0.001	1	0
1963	Mean patch area		Shannon diversity index		Hedgerow length		Number of disconnected networks of hedgerows	
Mean patch area	1	0						
Shannon diversity index	-0.59	0.01	1	0				
Hedgerow length	-0.58	0.01	0.12	0.63	1	0		
Number of disconnected networks of hedgerows	-0.8	<0.001	0.39	0.11	0.52	0.02	1	0

**Grasslands**								
2000	Shannon diversity index		Mean nearest Euclidean distance between grassland patches		Mean patch area		Grassland percentage	
	Coefficient	p	Coefficient	p	Coefficient	p	Coefficient	p
Shannon diversity index	1	0						
Mean nearest Euclidean distance between grassland patches	-0.14	0.6	1	0				
Mean patch area	-0.85	<0.001	0.068	0.8	1	0		
Grassland percentage	-0.27	0.3	-0.027	0.9	0.22	0.4	1	0
1985	Mean patch area		Grassland percentage		Shannon diversity index		Mean nearest Euclidean distance between grassland patches	
Mean patch area	1	0						
Grassland percentage	0.22	0.42	1	0				
Shannon diversity index	-0.84	<0.001	-0.54	0.03	1	0		
Mean nearest Euclidean distance between grassland patches	-0.084	0.77	-0.28	0.3	0.034	0.9	1	0
1963	Mean patch area		Grassland percentage		Shannon diversity index		Mean nearest Euclidean distance between grassland patches	
Mean patch area	1	0						
Grassland percentage	0.85	<0.001	1	0				
Shannon diversity index	-0.82	<0.001	-0.88	<0.001	1	0		
Mean nearest Euclidean distance between grassland patches	-0.59	0.02	-0.78	<0.001	0.6	0.02	1	0

Supplementary Table S2: Results of Spearman tests (coefficient and p-value) between landscape variables of each year used in linear models for birds

Birds												
2000												
	Mean nearest Euclidean distance between grassland patches		Mean patch area		Grassland percentage		Shannon diversity index		Hedgerow length		Number of disconnected networks of hedgerows	
	Coefficient	p	Coefficient	p	Coefficient	p	Coefficient	p	Coefficient	p	Coefficient	p
Mean nearest Euclidean distance between grassland patches	1	0										
Mean patch area	0.4	0.08	1	0								
Grassland percentage	-0.35	0.13	-0.26	0.28	1	0						
Shannon diversity index	-0.47	0.03	-0.86	<0.001	0.21	0.38	1	0				
Hedgerow length	-0.32	0.17	-0.74	<0.001	0.26	0.26	0.58		1	0		
Number of disconnected networks of hedgerows	-0.51	0.02	-0.81	<0.001	0.14	0.54	0.66	0.001	0.82	<0.001	1	0
1985												
	Mean nearest Euclidean distance between grassland patches		Mean patch area		Grassland percentage		Shannon diversity index		Hedgerow length		Number of disconnected networks of hedgerows	
Mean nearest Euclidean distance between grassland patches	1	0										
Mean patch area	0.086	0.7	1	0								
Grassland percentage	-0.26	0.26	-0.27	0.2	1	0						
Shannon diversity index	-0.093	0.7	-0.87	<0.001	0.078	0.7	1	0				
Hedgerow length	-0.2	0.4	-0.66	0.002	0.23	0.3	0.63	1	1	0		
Number of disconnected networks of hedgerows	-0.25	0.29	-0.72	<0.001	0.29	0.2	0.61	0.004	0.8	<0.001	1	0
1963												
	Grassland percentage		Mean patch area		Hedgerow length		Number of disconnected networks of hedgerows		Mean nearest Euclidean distance between grassland patches		Shannon diversity index	
Grassland percentage	1	0										
Mean patch area	0.57	0.009	1	0								
Hedgerow length	-0.1	0.6	-0.54	0.01	1	0						
Number of disconnected networks of hedgerows	-0.27	0.2	<0.001	<0.001	0.51	0.02	1	0				
Mean nearest Euclidean distance between grassland patches	-0.61	0.004	-0.46	0.04	0.45	0.04	0.2	0.3	1	0		
Shannon diversity index	-0.8	<0.001	-0.68	0.001	0.15	0.5	0.49	0.02	0.45	0.04	1	0

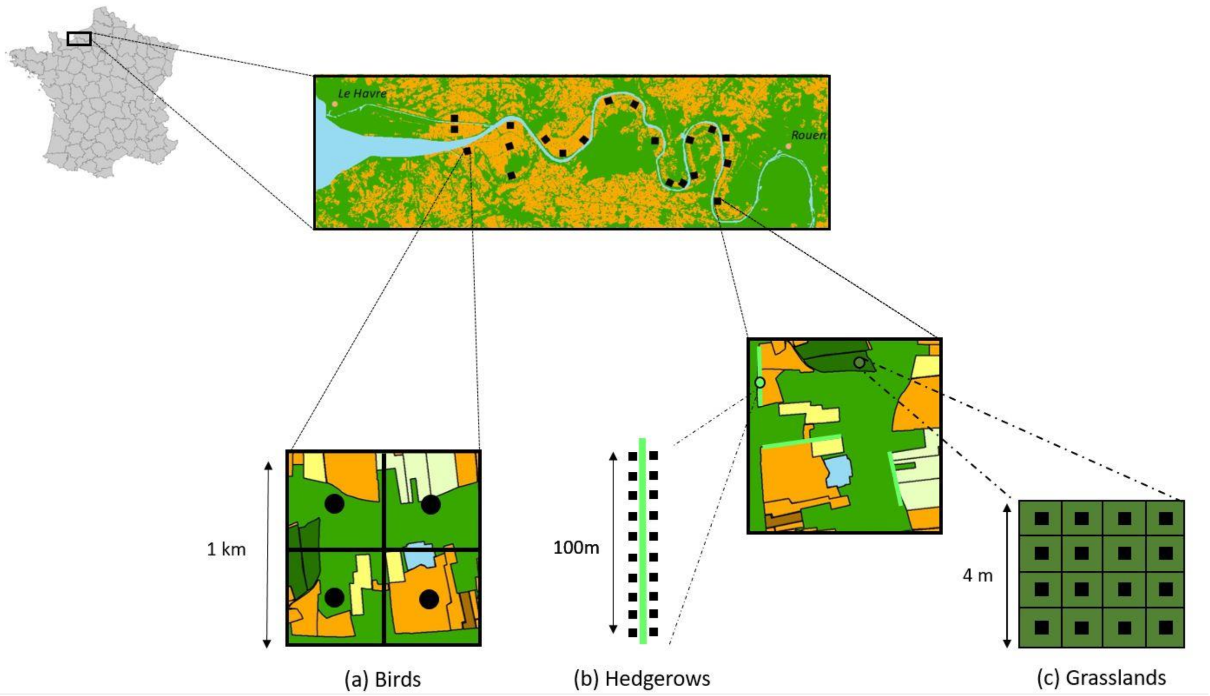

Supplementary Figure S4: Protocol of biological surveys conducted in the Seine valley in 2003. All biological surveys were conducted by Ernoult et al. in 2003. At each site : (a) 4 bird surveys spaced 500 meters apart were carried out with a total of 80 sampling points using the point-count method (Bibby et al. 2000). (b) 20 floristic surveys of hedgerows were conducted. A survey corresponds to 2 sections of 10m² divided into 10 quadrats of 1m x 1m on each side of the hedgerow. (c) 20 floristic surveys of grasslands were conducted. A survey corresponds to a 4m x 4m plot delimited at the center of the patch and divided into 16 quadrats where a sample of 0.20 x 0.20m was conducted. For both birds and plants, the analyses were based on one hand on the presence/absence of species and on the other hand the occurrence rate of species. This figure was created using ArcGIS Software (v. 10.6.1, URL: https://desktop.arcgis.com). The shapefile of the departments and the raster of land-uses were downloaded on the French government website (https://www.data.gouv.fr) and on the French Theia Land Data Centre (https://www.theia-land.fr) respectively.

Supplementary Table S3: List of all plant and bird species found across the 20 landscape windows along the Seine valley

**Hedgerows**	**Grasslands**	**Birds**
Achillea millefolium	Achilea millefolium	Acrocephalus palustris
Agrimonia eupatoria	Aegopodium podagraria	Acrocephalus schoenobaenus
Agropyrum caninum	Aethusia cynapium	Acrocephalus scirpaceus
Agropyrum repens	Agropyrum repens	Aegithalos caudatus
Agrostis canina	Agrostis capillaris	Alauda arvensis
Agrostis stolonifera	Agrostis stolonifera	Alcedo atthis
Alisma plantago aquatica	Alopecurus bulbosus	Anas crecca
Alliaria petiolata	Alopecurus geniculatus	Anas platyrhynchos
Allium triquetrum	Angelica sylvestris	Anthus pratensis
Allium vineale	Anthoxanthum odoratum	Anthus trivialis
Alopecurus geniculatus	Anthriscus sylvestris	Athene noctua
Anagallis arvensis	Anthyllis vulneraria	Buteo buteo
Angelica sylvestris	Apium nodiflorum	Carduelis cannabina
Anthoxanthum odoratum	Arabis hirsuta	Carduelis carduelis
Anthriscus sylvestris	Arrhenatherum elatius	Carduelis chloris
Arctium lappa	Atriplex prostrata	Certhia brachydactyla
Arrhenatherum elatius	Avenula pratensis	Cettia cetti
Artemisia vulgaris	Bellis perennis	Charadrius dubius
Arum maculatum	Brachypodium sylvaticum	Ciconia ciconia
Aster lanceolatus	bromus erectus	Circus cyaneus
Atriplex prostrata	Bromus mollis	Cisticola juncidis
Atropa bella donna	Bromus racemosus	Columba oenas
Avena fatua	Bromus sp	Columba palumbus
Barbarea vulgaris	Bromus sterilis	Corvus corone
Bellis perennis	Calystegia sepium	Coturnix coturnix
Berula erecta	Campanula rapunculus	Cuculus canorus
Bidens tripartita	Capsella bursa pastoris	Cygnus olor
Brachypodium sylvaticum	Cardamine pratensis	Dendrocopos major
Brassica nigra	Carduus crispus	Dendrocopos minor
Brassica olearaceaeX .colza.	Carex acutiformis	Dryocopus martius
Bromus inermis	Carex binervis	Emberiza cirlus
Bromus mollis	Carex diandra	Emberiza citrinella
Bromus racemosus	Carex distans	Emberiza schoeniclus
Bromus sterilis	Carex disticha	Erithacus rubecula
Bryona cretica	Carex flacca	Falco tinnunculus
Buddleja davidii	Carex hirta	Fringilla coelebs
Butomus umbellatus	Carex muricata	Fulica atra
Calamintha sylvatica	Carex ovalis	Gallinula chloropus
Caltha palustris	Carex otrubae	Garrulus glandarius
Calystegia sepium	Carex riparia	Hippolais polyglotta
Campanula rapunculus	Carex sp	Hirundo rustica
Capsella bursa.pastoris	Carex spicata	Larus ridibundus
Cardamine pratensis	Centaurea jacea	Locustella naevia
Carduus crispus	Centaurea nigra	Luscinia megarhynchos
Carex acutiformis	Cerastium arvense	Luscinia svecica
Carex diandra	Cerastium fontanum	Miliaria calandra
Carex disticha	Cerastium conglomeratus	Motacilla alba
Carex flacca	Cirsium arvense	Motacilla flava
Carex hirta	Cirsium vulgare	Muscicapa striata
Carex otrubae	convolvulus arvensis	Numenius arquata
Carex pseudocyperus	Crepis biennis	Parus caeruleus
Carex remota	Cynosurus cristatus	Parus major
Carex riparia	Dactylis glomerata	Parus montanus
Carex sp	Daucus carota	Parus palustris
Carex spicata	Deschampsia cespitosa	Passer domesticus
Centaurea jacea	Eleocharis palustris	Passer montanus
Centaurea nemoralis	Epilobium augustifolium	Perdix perdix
Centaurea nigra	Epilobium hirsutum	Phasianus colchicus
Centaurium pulchellum	Epilobium parviflorum	Phoenicurus ochruros
Cerastium fontanum	Epilobium tetragonum	Phoenicurus phoenicurus
Chenopodium album	Equisetum palustris	Phylloscopus collybita
Chenopodium polyspermum	Eupatorium cannabinum	Phylloscopus trochilus
Cirsium arvense	Euphorbia palustris	Pica pica
Cirsium acaule	Festuca arundinacea	Picus viridis
Cirsium palustre	Festuca ovina	Prunella modularis

Cirsium vulgare	Festuca pratensis	Pyrrhula pyrrhula
Cladium mariscus	Festuca rubra	Rallus aquaticus
Clematis vitalba	Festulolium	Saxicola rubetra
convolvulus arvensis	Ficaria ranuncoloides	Saxicola torquata
Crepis biennis	Fillipendula ulmaria	Serinus serinus
Cynosurus cristatus	Fraxinus excelsior	Sitta europaea
Dactylis glomerata	Galium aparine	Streptopelia decaocto
Daucus carota	Galium mollugo	Streptopelia turtur
Deschampsia cespitosa	Galium palustre	Sturnus vulgaris
Dipsacus fullonum	Galium verum	Sylvia atricapilla
Echinochloa crus galli	Gaudinia fragilis	Sylvia borin
Eleocharis palustris	Geranium dissectum	Sylvia communis
Epilobium hirsutum	Geranium molle	Tadorna tadorna
Epilobium palustre	Geranium pusillum	Troglodytes troglodytes
Epilobium parviflorum	Geranium robertianum	Turdus merula
Epilobium tetragonum	Glechoma hederacea	Turdus philomelos
Epipactis helleborine	Glyceria fluitans	Turdus viscivorus
Equisetum arvense	Glyceria maxima	Tyto alba
Equisetum palustris	Hedera helix	Vanellus vanellus
Equisetum pratense	Heracleum sphondylium	
Eupatorium cannabinum	Holcus lanatus	
Euphorbia palustris	Hordeum murinum	
Festuca arundinacea	Hordeum secalinum	
Festuca pratensis	Humulus lupulus	
Festuca rubra	Hydrocotyle vulgaris	
Ficaria ranunculoïdes	Hypochaeris radicata	
Filipendula ulmaria	Iris pseudacorus	
Fragaria vesca	Juncus acutiflorus	
Galinsoga parviflora	Juncus articulatus	
Galium aparine	Juncus effusus	
Galium mollugo	Juncus gerardii	
Galium palustre	Juncus inflexus	
Galeopsis angustifolia	Juncus subnodulosus	
Galeopsis tetrahit	Juncus sp	
Geranium dissectum	Lathyrus pratensis	
Geranium molle	Lolium multiflorum	
Geranium robertianum	Lolium perenne	
Geum urbatum	Lotus corniculatus	
Glechoma hederacea	Lotus pedunculatus	
Glyceria fluitans	Lychnis flo cuculi	
Gnaphalium uliginosum	Lycopus europaeus	
Hedera helix	Lysimachia numularia	
Heracleum sphondylium	Lysimachia vulgaris	
Holcus lanatus	Lythrum salicaria	
Hordeum secalinum	Medicago lupulina	
Humulus lupulus	Medicago sativa	
Hypericum perforatum	Mentha aquatica	
Hypericum quadrangulum	Mentha pulegium	
Impatiens capensis	Mentha sp	
Inula conyzae	Mentha spicata	
Iris foetidissima	Moehringia trinervia	
Iris pseudacorus	Myosotis arvensis	
Juncus acutiflorus	Myosotis discolor	
Juncus articulatus	Myosotis scorphioides	
Juncus bufonius	Oenanthe fistulosa	
Juncus effusus	Oenanthe salaifolia	
Juncus inflexus	Persicaria maculosa	
Lamiastrum galeobdolon	Phalaris arundinacea	
Lamium album	Phleum pratense	
Lamium purpureum	Phragmites australis	
Lapsana communis	Plantago lanceolata	
Lathyrus pratensis	Plantago major	
Leontodon hispidus	Plantago minor	
Leucanthemum vulgare	Poa pratensis	

Lolium multiflorum	Poa trivialis	
Lolium perenne	Polygonum amphibium	
Lonicera periclymenum	Polygonum aviculare	
Lotus corniculatus	Polygonum hidropiper	
Lotus pedunculatus	Polygonum sp	
Lychnis flos cuculi	Potentilla anserina	
Lycopus europaeus	Potentilla reptans	
Lysimachia nummularia	Prunella vulgaris	
Lysimachia vulgaris	Quercus petraea	
Lythrum salicaria	Ranunculus acris	
Matricaria chamomilla	Ranunculus flammula	
Matricaria discoidea	Ranunculus repens	
Medicago arabica	Ranunculus sardous	
Medicago lupulina	Rubus fruticosus	
Melilotus altissimus	Rubus sp	
Melilotus officinalis	Rumex acetosa	
Mentha aquatica	Rumex conglomeratus	
Mentha arvensis	Rumex crispus	
Mercurialis perennis	Rumex obtusifolius	
Moehringia trinervia	Samolus valerandi	
Mycelis muralis	scrophularia auriculata	
Myosotis arvensis	Senecio vulgaris	
Myosotis scorphioides	Silene latifolia	
Myosoton aquaticum	Solanum dulcamaria	
Oenanthe fistulosa	Sonchus arvensis	
Ononis spinosa	Sonchus asper	
Papaver rhoeas	Sonchus sp	
Persicaria persicaria	Stellaria media	
Petasites hybridus	Symphitum officinale	
Phalaris arundinacea	Taraxacum sp.	
Phleum pratense	Thalictrum aquilegifolium	
Phragmites australis	Thalictrum flavum	
Picris hieracioides	Tragopogon sp	
Plantago lanceolata	Trifolium campestre	
Plantago major	Trifolium dubium	
Poa nemoralis	Trifolium pratense	
Poa pratensis	Trifolium repens	
Poa trivialis	Urtica dioica	
Polygonum aviculare	Veronica hederifolia	
Polygonum hydropiper	Veronica sp	
Polygonum persicaria	Veronica verna	
Polygonum sp	Vicia cracca	
Potentilla anserina	Vicia sativa	
Potentilla reptans	Vicia sepium	
Primula veris		
Prunella vulgaris		
Pulicaria dysenterica		
Ranunculus acris		
Ranunculus aquatilis		
Ranunculus flammula		
Ranunculus repens		
Rorippa amphibia		
Rorripa nasturtium aquaticum		
Rorippa palustris		
Rosa sp		
Rubus fruticosus		
Rubus sp		
Rumex acetosa		
Rumex conglomeratus		
Rumex crispus		
Rumex hydrolaphatum		
Rumex obtusifolius		
Rumex sp		

Samolus valerandi		
Scirpus maritimus		
scrophularia auriculata		
Scrophularia nodosa		
Scutellaria galericulata		
Senecio erucifolius		
Senecio vulgaris		
Setaria verticillata		
Silene latifolia		
Silene vulgaris		
Sisymbrium officinale		
Solanum dulcamaria		
Solanum nigrum		
Sonchus arvensis		
Sonchus asper		
Stachys palustris		
Stachys sylvatica		
Stellaria graminea		
Stellaria media		
Symphitum officinale		
Synapsis arvensis		
Tamus communis		
Taraxacum officinale		
Thalictrum aquilegifolium		
Thalictrum flavum		
Torilis japonica		
Tragopogon pratensis		
Trifolium dubium		
Trifolium pratense		
Trifolium repens		
matricaria maritima inodora		
Trisetum flavescens		
Triticum aestivum		
Urtica dioica		
Valeriana officinalis		
Verbena officinalis		
Veronica anagalis aquatica		
Veronica beccabunga		
Veronica catenata		
Veronica chamaedrys		
Veronica scutellata		
Veronica hederafolia		
Veronica serpyllifolia		
Veronica persica		
Veronica sp		
Vicia cracca		
Viola canina		
Viola reichenbachiana		
Viola sp		

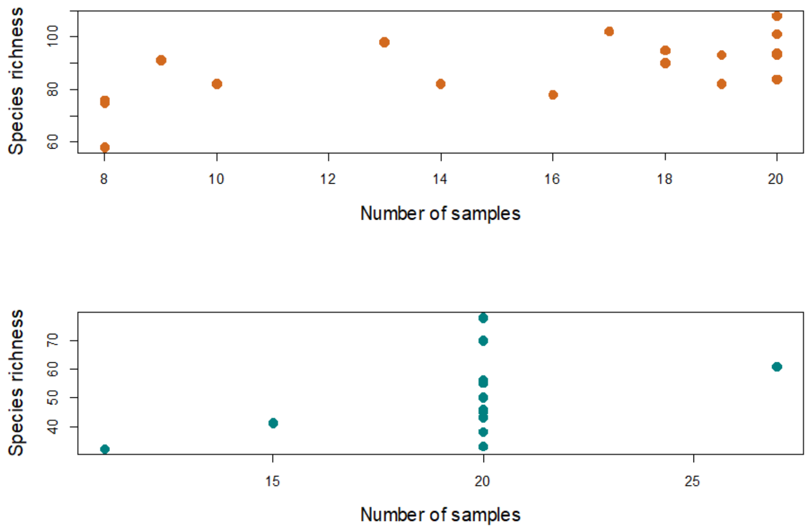

Supplementary Figure S5: Rarefaction curves of hedgerow and grassland assemblages. Within each landscape window, from 12 to 27 floristic surveys of grasslands, and from 8 to 20 floristic surveys of hedgerows were conducted. These plots represent the number of species of hedgerow and grassland plants as a function of the number of samples. They were represented to ensure that number of samples did not have a major influence on the species richness quantified in each landscape window. This figure was created using R Software (v. 4.02, URL: https://www.r-project.org).

Supplementary Table S4: Results of Spearman tests (coefficient and p-value) on functional traits of hedgerows, grasslands and birds.

Hedgerows	Seed mass		Allocation		Beginning of flowering		Flowering duration		Lifespan	
	Coefficient	p	Coefficient	p	Coefficient	p	Coefficient	p	Coefficient	p
Seed mass	1	0								
Allocation to clonal reproduction	-0.24	<0.001	1	0						
Beginning of flowering	-0.02	0.81	-0.04	0.59	1	0				
Flowering duration	-0.14	0.04	-0.19	<0.01	-0.26	<0.001	1	0		
Lifespan	-0.14	0.04	0.52	<0.001	-0.03	0.70	-0.34	<0.001	1	0

Grasslands	Seed mass		Allocation		Beginning of flowering		Flowering duration		Lifespan	
	Coefficient	p	Coefficient	p	Coefficient	p	Coefficient	p	Coefficient	p
Seed mass	1	0								
Allocation to clonal reproduction	-0.18	0.02	1	0						
Beginning of flowering	-0.044	-0.044	-0.22	<0.01	1	0				
Flowering duration	-0.035	0.66	0.11	0.16	-0.3	<0.001	1	0		
Lifespan	-0.17	0.03	0.38	<0.001	-0.38	<0.001	0.22	<0.01	1	0

Birds	Body mass		Egg number		Beginning of breeding		Number of breeding events		Lifespan	
	Coefficient	p	Coefficient	p	Coefficient	p	Coefficient	p	Coefficient	p
Body mass	1	0								
Egg number	-0.14	0.22	1	0						
Beginning of breeding	-0.23	0.04	-0.19	0.1	1	0				
Number of breeding events	-0.29	0.01	-0.3	<0.01	0.32	<0.01	1	0		
Lifespan	0.7	<0.001	-0.09	0.43	-0.29	<0.01	-0.27	0.02	1	0

Supplementary Table S5: Significance of the phylogenetic signals of the 5 continuous traits of plants and birds. Blomberg's K values vary from 0 (no signal) to infinity. P-values estimated from randomization testing, while shuffling 999 times the names of the species on the phylogenetic tree.

Traits	Hedgerows		Grasslands	
	Blomberg	p-value	Blomberg	p-value
Seed mass	0.1	0.003	0.7	0.001
Allocation to clonal reproduction	0.01	0.03	0.01	0.2
Beginning of flowering	0.02	0.009	0.02	0.04
Flowering duration	0.007	0.3	0.007	0.7
Lifespan	0.01	0.08	0.02	0.07

Traits	Birds	
	Blomberg	p-value
Body mass	0.5	0.001
Egg number	0.9	0.001
Beginning of breeding	0.3	0.05
Number of breeding events	0.5	0.001
Lifespan	0.9	0.001

Supplementary Table S6: Results of Spearman tests (coefficient and p-value) between species richness and CMVs of each biological model.

	Species richness	
	Coefficent	p
Hedgerows		
- CWV of seed mass	0.52	0.02
- CWV of onset of flowering	-0.32	0.19
- CWV of flowering duration	-0.19	0.46
- CWV of lifespan	-0.21	0.4
Grasslands		
- CWV of seed mass	0.66	0.007
- CWV of allocation to clonal reproduction	-0.72	0.002
- CWV of onset of flowering	-0.14	0.6
- CWV of lifespan	-0.003	0.9
Birds		
- CWV of body mass	0.43	0.053
- CWV of beginning of breeding	0.4	0.08
- CWV of number of breeding events	-0.28	0.22

Supplementary Table S7: Results of Spearman tests (coefficient and p-value) between the percentage of crops and the percentage of grasslands for the three date studied.

	Current	1985	1963
Correlation coefficient	-0.84	-0.78	-0.61
p-value	<0.001	<0.001	0.004

Supplementary Table S8: Results of the variance inflation factor (VIFs) of all linear models conducted.

	Models	VIF
Hedgerows	Functional indices of hedgerow ~ Current Shannon + Current Area mean + Current hedgerows lenght + Current Networks + 15 years ago Shannon + 15 years ago Area mean + 15 years ago hedgerows lenght + 15 years ago Networks 40 years ago Shannon + 40 years ago Area mean + 40 years ago hedgerows lenght + 40 years ago Networks	54
	Functional indices of hedgerows ~ Current Shannon + Current Area mean + Current hedgerows lenght + Current Networks	8.24
	Functional indices of hedgerows ~ 15 years ago Shannon + 15 years ago Area mean + 15 years ago hedgerows lenght + 15 years ago Networks	7.04
	Functional indices of hedgerows ~ 40 years ago Shannon + 40 years ago Area mean + 40 years ago hedgerows lenght + 40 years ago Networks	5.95
Grasslands	Functional indices of grasslands ~ Current Shannon + Current Area mean + Current grassland percentage + Current mean distance + 15 years ago Shannon + 15 years ago Area mean + 15 years ago grasslands percentage + 15 years ago mean distance + 40 years ago Shannon + 40 years ago Area mean + 40 years ago hedgerows lenght + 40 years ago Networks	103
	Functional indices of grasslands ~ Current Shannon + Current Area mean + Current grassland percentage + Current mean distance	4.3
	Functional indices of grasslands ~ 15 years ago Shannon + 15 years ago Area mean + 15 years ago grasslands percentage + 15 years ago mean distance	4.79
	Functional indices of grasslands ~ 40 years ago Shannon + 40 years ago Area mean + 40 years ago grasslands percentage + 40 years ago mean distance	7.63
Birds	Functional indices of birds ~ Current Shannon + Current Area mean + Current grassland percentage + Current mean distance + Current hedgerows lenght + Current Networks + 15 years ago Shannon + 15 years ago Area mean + 15 years ago grasslands percentage + 15 years ago mean distance + 15 years ago hedgerows lenght + 15 years ago Networks + 40 years ago Shannon + 40 years ago Area mean + 40 years ago grasslands percentage + 40 years ago mean distance + 40 years ago hedgerows lenght + 40 years ago Networks	2971
	Functional indices of birds ~ Current Shannon + Current Area mean + Current grassland percentage + Current mean distance + Current hedgerows lenght + Current Networks	10
	Functional indices of birds ~ 15 years ago Shannon + 15 years ago Area mean + 15 years ago grasslands percentage + 15 years ago mean distance + 15 years ago hedgerows lenght + 15 years ago Networks	6.98
	Functional indices of birds ~ 40 years ago Shannon + 40 years ago Area mean + 40 years ago grasslands percentage + 40 years ago mean distance + 40 years ago hedgerows lenght + 40 years ago Networks	8.4
